# Normalized long read RNA sequencing in chicken reveals transcriptome complexity similar to human

**DOI:** 10.1186/s12864-017-3691-9

**Published:** 2017-04-24

**Authors:** Richard I. Kuo, Elizabeth Tseng, Lel Eory, Ian R. Paton, Alan L. Archibald, David W. Burt

**Affiliations:** 1 The Roslin Institute and Royal (Dick) School of Veterinary Studies, University of Edinburgh, Easter Bush, Midlothian, EH25 9RG UK; 2 Pacific Biosciences, Menlo Park, CA USA; 3 The University of Queensland, St. Lucia, Canberra, QLD 4072 Australia

**Keywords:** Iso-Seq, PacBio, Single molecule long read sequencing, Transcriptome sequencing, RNAseq, Chicken, Avian, Gallus gallus, Genome annotation, Coding RNA, Non-coding RNA

## Abstract

**Background:**

Despite the significance of chicken as a model organism, our understanding of the chicken transcriptome is limited compared to human. This issue is common to all non-human vertebrate annotations due to the difficulty in transcript identification from short read RNAseq data. While previous studies have used single molecule long read sequencing for transcript discovery, they did not perform RNA normalization and 5′-cap selection which may have resulted in lower transcriptome coverage and truncated transcript sequences.

**Results:**

We sequenced normalised chicken brain and embryo RNA libraries with Pacific Bioscience Iso-Seq. 5′ cap selection was performed on the embryo library to provide methodological comparison. From these Iso-Seq sequencing projects, we have identified 60 k transcripts and 29 k genes within the chicken transcriptome. Of these, more than 20 k are novel lncRNA transcripts with ~3 k classified as sense exonic overlapping lncRNA, which is a class that is underrepresented in many vertebrate annotations. The relative proportion of alternative transcription events revealed striking similarities between the chicken and human transcriptomes while also providing explanations for previously observed genomic differences.

**Conclusions:**

Our results indicate that the chicken transcriptome is similar in complexity compared to human, and provide insights into other vertebrate biology. Our methodology demonstrates the potential of Iso-Seq sequencing to rapidly expand our knowledge of transcriptomics.

**Electronic supplementary material:**

The online version of this article (doi:10.1186/s12864-017-3691-9) contains supplementary material, which is available to authorized users.

## Background

Transcriptome annotation is crucial for a wide array of biological research areas, including genomics, proteomics, epigenetics, immunology, and phylogenomics [1]. The identification of the full repertoire of transcribed elements provides information on the functional roles and relationships of genomic loci which in turn can be compared to understand a vast array of biological mechanisms. However, due to the complexity of transcript splicing and the limitations of previous technologies, researchers had to choose between low-throughput, costly methods to generate accurate full-length transcript models, such as cDNA cloning [2] or high-throughput, cheaper methods to generate imprecise transcript models, such as short read RNA sequencing [3, 4]. The current status of chicken annotation represents a prime example of this trade off.

The Ensembl chicken annotation (release 83), built primarily on short read RNAseq and comparative data, contains 17,108 genes with 17,954 transcripts [5]. These numbers stand out for two major reasons. The first reason is that the number of genes is far lower than that found for other vertebrate organisms, for example, the Ensembl (release 83) human annotation contains 60,675 genes (including coding and non-coding genes). The difference in the number of genes annotated in the chicken and human genomes is heavily influenced by lack of long non-coding gene predictions in the chicken annotation. While it can be argued that this may represent differences between mammals and birds, evidence that many more genes exist in birds can be seen in the cDNA support track on Ensembl. The second reason is that the current chicken annotation is almost entirely comprised of protein coding genes for which a single transcript is described. Again this is contrary to what we know from other vertebrates with the human annotation (Ensembl release 83) containing 199,184 transcripts (i.e. an average of 3.3 transcripts per gene). These discrepancies highlight major limitations to using short read RNA sequencing and comparative data for building gene and transcript models.

**Fig. 1:**
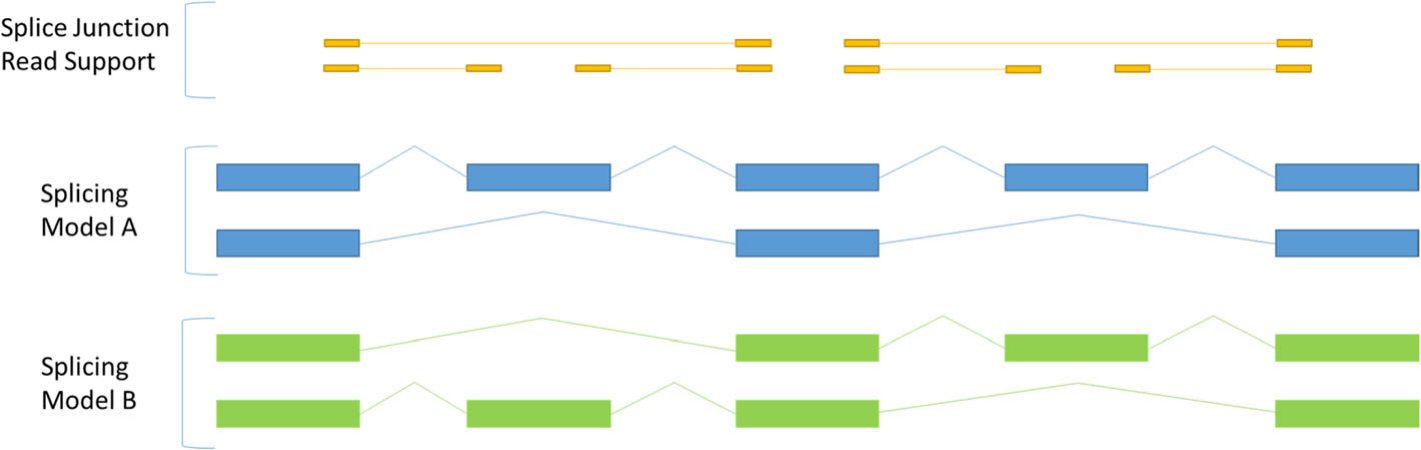
Short read transcript modelling problem. Example of transcript model that is impossible to resolve using short read data. Given the read support in *yellow*, it is impossible to determine which splicing model is real

With short read RNAseq data there are three major transcription characteristics that are difficult to determine [6]: (i) transcript start sites (TSS) and transcript termination sites (TTS), (ii) exon chaining, and (iii) transcriptional noise. If multiple TSS or TTS exist for a transcribed locus, then interior TSS and TTS can go undetected due to combinations of inconsistent read coverage, overlapping exons, and overlapping splice junctions. Thus for any transcript model produced via short read data, we often cannot determine if there are alternative TSS and TTS which have not been detected. Similarly, the process of chaining exons and splice junctions together to reconstruct full-length transcript sequences can be problematic. Since a single short read cannot usually span all splice junctions within a multiple splice junction transcript, transcript assemblers must predict which exons are linked to reconstruct the full length sequence. However, non-uniform transcript coverage can obscure the underlying model by suggesting different splicing events. Even with uniform read coverage, there are scenarios where the problem of identifying the correct exon chaining model is intractable (Fig. 1). The third issue with short read RNAseq data arises from transcriptional noise. Transcriptional noise becomes problematic when it occurs within intronic or intergenic regions. The origin of these reads is unclear and transcript assemblers have taken different approaches to minimizing the influence of transcriptional noise [7–9]. Despite these efforts, there are some clear implications of the phenomena. For example, due to the possible occurrence of transcriptional noise within intronic regions, it is difficult to determine if a transcript model should include a retained intron or not. When transcriptional noise occurs in intergenic regions it can be erroneously predicted as a gene or it can be fused with a neighbouring gene. When combining these three issues, the uncertainty of short read assembled transcript models becomes restrictive.

The annotation of most vertebrate genome sequences, except human and mouse, has been hampered by the lack of full length cDNA/transcript sequences for the species of interest and has instead had to largely rely upon Expressed Sequence Tags (ESTs) and their abundant successors, short read RNA-seq. As a result, the complexity of transcription of the chicken genome is underrepresented in the current genome annotation and constrains some analyses. For example, many differential expression analysis experiments rely on the annotation to define transcription events. Since a large number of alternative transcript models are likely missing in many vertebrate annotations, alternative transcription dependent mechanisms may have been unknowingly omitted from these studies.

While these issues are common in short read RNAseq data, they are practically eliminated with long read sequencing where the full-length of a transcript may be sequenced in a single read. With full-length sequencing, TSS and TTS can be easily defined since the reads span the entire length of the transcript. Similarly, predicting exon chaining from probabilistic models is not necessary. Transcriptional noise is reduced and in the cases where it does occur, it is more easily identified.

With the recent development of Pacific Biosciences (PacBio) SMRT Iso-Seq sequencing [10], it is now possible to attain high throughput, full-length transcript sequencing. While this technology has huge potential for transcriptome annotation, it still requires development for both library preparation and data analyses. Iso-Seq has been used in previous studies to identify transcript sequences [11–14], however, there are two main issues with these earlier approaches. The first issue is that normalization of the RNA libraries was not performed, thus many low abundance transcripts may not have been sequenced due to the higher probability for attaining reads from high abundance transcripts. The second issue is that transcription start sites could not be confirmed due library preparation protocols lacking 5′-cap capture, thus the identified transcript sequences are not guaranteed to be full-length.

To address these concerns, we generated PacBio SMRT Iso-Seq sequencing data from chicken brain and embryo RNA. Both RNA libraries were normalized to reduce over-represented transcripts, however we only performed 5′ cap selection on the embryo library. We also performed Illumina short read RNA sequencing on 20 tissue types to both verify transcribed loci and compare transcript models.

We identified important considerations for Iso-Seq sequencing and data analyses. Using this understanding of the data limitations, we surveyed the chicken transcriptome to discover transcriptional complexity similar to the human annotation. This complexity is comprised of the type and number of alternative transcription events, previously unannotated biotypes in chicken, and transcriptional sequence variance between species. We have also identified two classes of long non-coding RNA that are under-represented in all mammalian annotations.

Our results provide guidance for future Iso-Seq studies as well as insight into chicken and all vertebrate transcriptomes. The data from this study were submitted to the European Nucleotide Archive (ENA) and used by Ensembl for their future chicken annotations.

## Results

### Processing PacBio data to create a high quality non-redundant PacBio transcriptome

#### Strategy for processing of PacBio Iso-Seq reads

**Fig. 2:**
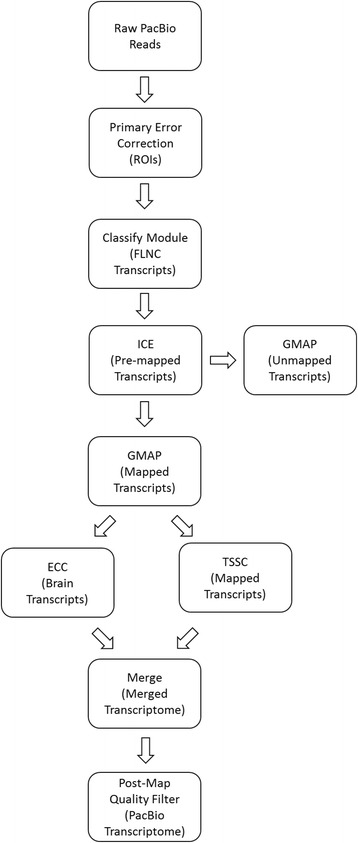
Full pipeline for processing PacBio data

Analysing PacBio Iso-Seq data requires a very different approach as compared to short read RNAseq data. Initial processing of this type of data focuses on reducing the final error rate of the acquired transcript sequences. While the raw error rate of PacBio sequencing is around 11-14% [10], the use of circular sequencing and computational error correction can greatly increase the final quality score. The software for achieving this is still in an early stage of development and evolving rapidly. We have adopted methodology supported by the PacBio development team known as the Iso-Seq pipeline, also known as the *pbtranscript-tofu* analysis suite [15], and incorporated it into our own pipeline (Fig. 2). The methods used to error correct Iso-Seq reads can have major implications for the limitations of downstream analyses. We have identified some major considerations when processing this type of data.

#### Raw data error correction

We attained 805,606 reads-of-insert (ROIs) from the brain and 247,626 ROIs from the embryo libraries. The lower yield for the embryo project was a result of issues with loading SMRT cells with a size selection of lower than 1-kb. Graphs for the read lengths for each size selection are available in additional files (see Additional file 1). Since every ROI sequence should begin with the adapter sequence, we calculated the quality scores for each ROI by aligning the known adapter sequence to the adapter sequence within each ROI sequence and dividing the number of matches in the alignment by the length of the adapter sequence (see Methods). ROI average quality scores were 96.1% for brain 1-kb, 95.4% for brain 2-kb, 84.7% for embryo 0.8-kb, and 85.9% for embryo 2-kb. We ran *pbtranscript-tofu pbclassify* [15] with the ROIs as input to attain 515,175 full-length, non-chimeric (FLNC) transcripts for brain and 138,266 FLNC transcripts for embryo. After a further round of error correction using Iso-Seq iterative clustering for error correction (ICE) tool, from the *pbtranscript-tofu* analysis suite [15], we attained 211,292 transcripts for brain and 14,776 transcripts for embryo.

We mapped the resulting transcripts sequences to the Gallus_gallus_4 genome assembly using GMAP [16]. 199,560 transcripts from brain and 11,881 transcripts from embryo mapped to this genome assembly. This left 11,732 brain and 3028 embryo transcripts unmapped. The unmapped transcripts are most likely a combination of transcripts which should map to the unassembled regions of the genome and transcripts which contain large errors missed in the previous filtering steps.

#### Collapsing transcript models to reduce redundancy

**Fig. 3:**
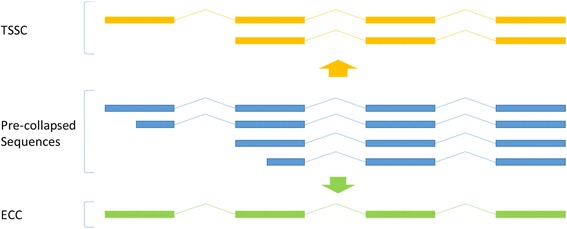
Iso-Seq mapped read collapsing methods. Two methods for collapsing PacBio mapped sequences to remove redundant models: Transcription Start Site Collapse (TSSC) and Exon Cascade Collapse (ECC). ECC is more aggressive in collapsing

In previous studies [11–14], no 5′ cap selection was performed, thus possible 5′ degradation was ignored. In order to understand if the absence of 5′-cap selection in the library preparation would result in significant loss of TSS in our final transcript models, we collapsed the mapped transcript sequences from both the brain and embryo libraries using two methods from the PacBio *pbtranscript-tofu* analysis suite [15]. Both methods assume that the 3′ end is intact, thus any transcript models with unique TTS are not collapsed. In the first method, termed Transcription Start Site Collapse (TSSC) (Fig. 3), transcripts with identical splice junctions and 3′-ends but varying TSS are collapsed so that only the longest transcript is kept. While this method is inappropriate for libraries which were not 5′-cap selected, we still use the results from TSSC as a comparison. The second method, termed Exon Cascade Collapse (ECC) (Fig. 3), is identical to the first except that transcripts that are missing 5′ end exons are included in the collapsing group. ECC is a more aggressive form of collapsing than TSSC and all transcripts that would be collapsed in TSSC would also be collapsed in ECC.

We looked at the ratio of the pre-collapsed transcripts to the collapsed transcript for each library from each collapsing method as an indicator of 5′-sequence loss. Since there should be no 5′ sequence loss for the embryo library, the ratio of pre-collapsed to collapsed transcripts in the embryo library is used as a baseline for this comparison.

From 199,560 brain transcripts after running ICE, we attained 80,814 TSSC and 55,932 ECC models. From 11,881 embryo transcripts after running ICE, we attained 9368 TSSC and 8468 ECC models. Thus the number of transcript models drop by 59.5 and 72.0% for TSSC and ECC methods with the brain data, whereas the embryo transcript number only decreased by 21.2 and 28.7%, respectively. It is possible that these differences are caused by real biological differences in transcription start sites, however, 5′-sequence loss seems more likely and should not be ignored in the downstream analyses.

To reduce redundancy in our dataset we used the TSSC method for the embryo sequences and ECC for the brain sequences. This resulted in 55,932 transcripts from brain and 9368 transcripts from embryo after collapsing. Although it might seem strange for there to be a significant amount of collapsing within the embryo data there are biological reasons for this to occur. For instance, TSS are known to be variable so that otherwise identical transcripts can have different TSS, as shown by the evidence of wide promoter regions from cap analysis of gene expression (CAGE) studies [17]. While it is possible that using the TSSC method for the embryo data can result in the loss of unique transcript models that represent different TSS, due to the low coverage (compared to short read RNAseq data) for each transcript, it is impossible to identify whether differences in the observed TSS are a result of a single wide promoter region or from multiple distinct promoter regions. For the purposes of this study, we chose to follow a conservative approach which meant removing possibly redundant information at the cost of filtering out some real biological information.

#### Filtering out low quality models using post-mapping quality estimates

**Fig. 4:**
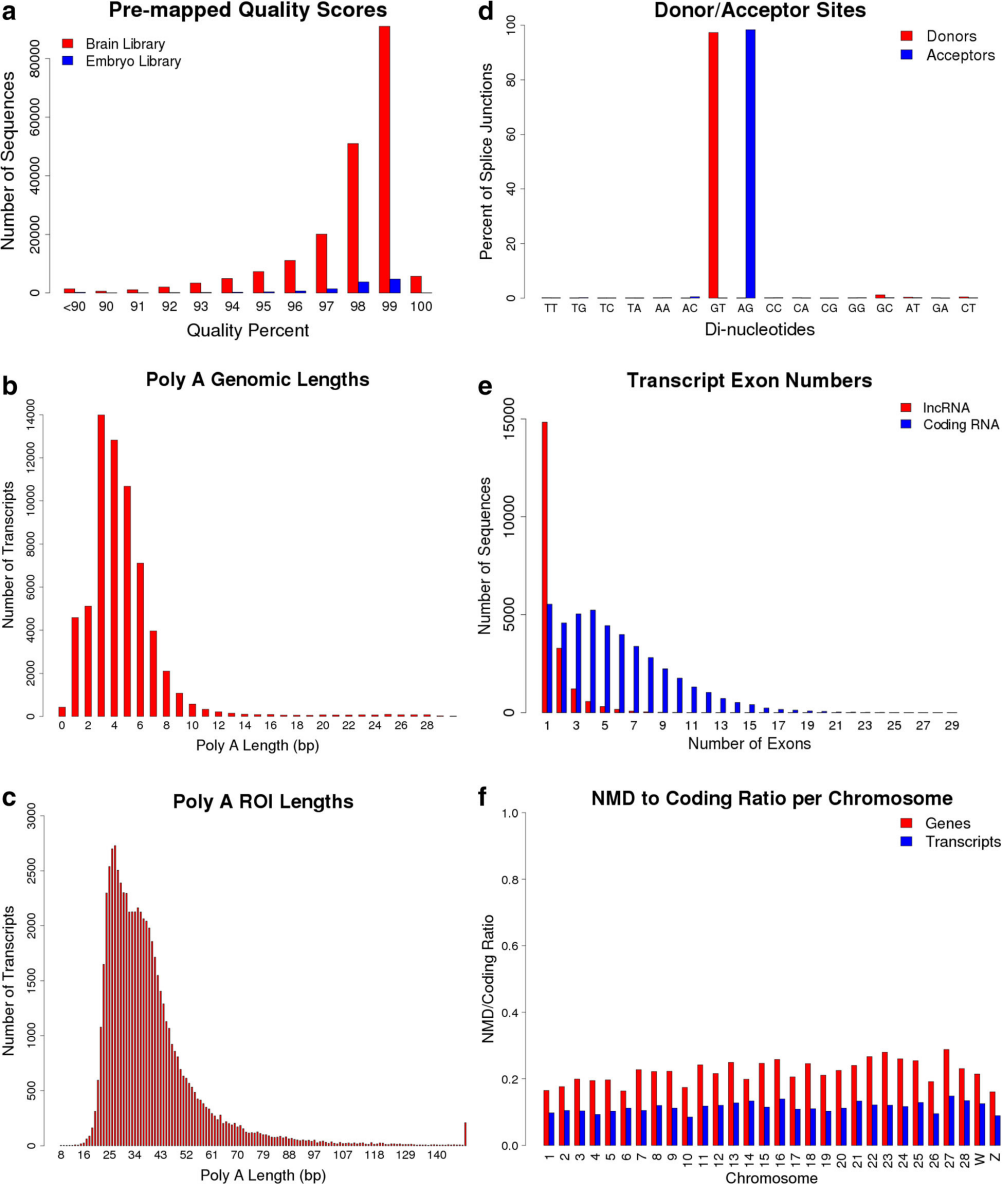
Analyses of PacBio sequencing **a** Quality scores of PacBio sequence before mapping to the genome. **b** Length of genomic Poly A’s downstream of PacBio mapped models. **c** Length of Poly A tails in ROI sequences. **d** Intronic Donor/Acceptor sites. **e** Number of exons per transcript for coding and lncRNA transcripts. **f** NMD to coding transcript ratio per chromosome in PacBio transcriptome

Mapping the transcript sequences to the genome can be seen as a final error correction step. The differences between the pre-mapped and post-mapped sequences provide an indication of the error rate for the sequences after all prior error correction and allows for the filtering of erroneous models that are a result of poor mapping. Post-mapped sequences are defined by using the genomic nucleotides for the predicted genomic coordinates of the mapped transcripts. True sequence variation can contribute to sequence differences, however the primary purpose of this transcriptome annotation is to identify models based on the reference genome. For each transcript sequence, we aligned the pre-mapped sequence with the post-mapped sequence and counted the number of mismatches within the alignment. We calculated the error rate by dividing the number of mismatches by the length of the transcript. Using this method, we selected for transcript sequences with less than 10% error rate. This resulted in 55,315 brain transcripts and 9206 embryo transcripts, greater than 98% retention for both sample types (Fig. 4a).

While a 10% error rate may seem high in comparison to short read data, the distribution of transcripts based on quality scores/error rates shows that the mode is 99% quality score. In addition, we are able to attain unique mappings due to the length of the sequences. Thus while the error rate makes this data unsuitable for polymorphism detection, it is low enough to provide accurate transcript models. There are several possible explanations for the occurrence of transcripts that did not meet our 10% error rate threshold. The observed discrepancy between the mapped and pre-mapped sequences could have resulted from sequencing error, errors in the reference genome assembly, and/or biological differences due to the difference between the genomes of the reference assembly specimen (red jungle fowl) and the chickens sampled in this project. Due to the ambiguity of the source of error, we chose to remove these sequences from our downstream analyses. Previous studies did not report using this method of error correction which may indicate that some of their transcript models contained erroneous models [13, 14].

#### Identifying possible transcript truncation due to internal poly-A regions

Poly-A tail selection is a commonly used method of attaining transcript sequences with intact 3′-ends [11, 12]. However, internal stretches of A’s can bind to oligo-dT primers thus resulting in a 3′ truncated transcript sequence. In short read RNAseq sequencing this usually does not pose a large problem because many inserts will be sequenced and the farthest downstream 3′-end will usually be selected as the TTS. However, with single molecule long read sequencing, we make the assumption that each sequence has an intact 3′-end. To assess whether this is an appropriate assumption, we investigated the possible rate of occurrence of poly-A truncation by looking at the 3′-genomic sequence of each predicted transcript. The primers used for poly-A tail selection were designed to bind to a minimum of a stretch of 20 A’s. Due to the prevalence of insertion/deletion sequencing errors in PacBio sequencing we used a 30 bp window. Thus for each PacBio transcript, we extracted the 30 bp downstream genomic sequence and looked for stretches of A’s. If a PacBio transcript model were a result of internal poly-A truncation, we should see a stretch of at least 20 A’s within this region. We allowed one mismatch within a string of A’s and used the longest string of A’s for our calculations. From the 64,277 identified transcripts, only 700 had a stretch of at least 20 A’s immediately following their putative TTS in the genome sequence. Thus, around 1.1% of the deduced transcripts may be artificially truncated (Fig. 4b).

We also looked at the length of poly-A’s within the respective ROI’s as a comparison (The poly-A tails within the ROI sequences are removed during the *pbtranscript-tofu pbclassify* error correction step). If the length of poly-A’s in the ROI’s (Fig. 4c) are much longer than the length of internal poly-A’s (Fig. 4b) then the prevalence of internal poly-A truncation is likely to be minimal. The ROI’s have an average of 39.6 consecutive A bases in their poly-A tails and a peak at about 27 bp (Fig. 4c). This matches a previous study that found a peak of TAIL-seq tags with poly-A tails of about 20 nt in length [18]. Due to the dominance of true poly-A tails at around 27 bp it is non-trivial to differentiate transcripts with real poly-A tails from those that may be truncated due to internal poly-A stretches. However, since only a maximum of 1.1% of the transcript models could have internal poly-A truncation, this issue seems to have a limited effect on Iso-Seq data. This methodology for identifying possibly truncated transcripts can be used in future Iso-Seq studies to flag and/or filter transcript models.

#### Verifying canonical splice sites

We looked at splice site donor and acceptor intronic dinucleotides to see if they conformed to canonical observations [19]. The GT-AG donor-acceptor sequence was used by 97.0% of brain transcripts and 98.0% of embryo transcripts, both very similar to the reported 98.7% in mammals [19] (Fig. 4d). While GMAP does have a bias for mapping splice sites to conform to the canonical GT-AG scenario, the similarity between the canonical splice site percentages suggests that the splice site predictions are generally accurate.

#### Merging the transcriptomes from each sample

We merged the brain and embryo transcripts to form a unified transcriptome annotation to be used for further analyses. Merging was performed with the criteria that transcripts were merged if they had the same exon structures with less than a 10-bp difference for each exon start/end and less than 20-bp difference for the transcript start/end. We allowed for slight differences in exon start/end and transcript start/end to account for possible mapping errors as a result of insertion/deletion sequencing errors which are the most prevalent in Iso-Seq sequencing. When merging, the transcript with the earlier start site was used as the new representative transcript. This merging process resulted in a total of 64,277 distinct transcripts. Only 488 transcripts between the two sets were merged which translates to 244 shared transcripts. Of the 244 shared transcripts, 176 had the brain transcript as the new representative model, meaning that for this set, the brain transcripts had an earlier TSS.

We grouped the transcripts into putative gene models by clustering transcripts that had at least a one nucleotide overlap. This resulted in 29,013 putative genes which we will refer to simply as genes. Of these, 4579 genes had transcripts from both brain and embryo libraries with only 621 genes having only transcripts from embryo libraries. This indicates that while most genes are transcribed across sample types, the resulting transcripts differ. Thus providing more support for the observation that alternative transcription plays a significant role in tissue differentiation [20]. However, due to the lack of 5′ cap selection for the brain dataset, there may be more shared transcripts than we observed simply because we lacked the 5′ end of the brain transcripts. Since short read data is generally inaccurate with respect to isoform level quantification, this biological phenomenon would be very difficult to detect without long read sequencing.

#### Estimating gene numbers for unmapped transcripts

Since we were unable to use genomic locations to group the unmapped transcripts, we instead used the BLASR [21] mapper to find hits between the unmapped reads. Reads were grouped if they had same stranded BLASR hits. 11,732 unmapped reads from the brain and 3028 unmapped reads from the embryo were clustered into 8812 groups. This indicates a significant number of genes that are not currently represented in the Chicken annotations due to gaps in the genome assembly. We excluded these unmapped transcripts from further analyses due to the uncertainty of the sequence quality and the effects that would have on the prediction methods we used.

### Comparison with previous chicken PacBio transcriptome sequencing studies

In order to estimate the benefit of library normalization with respect to the efficiency of transcriptome coverage for each SMRT cell used, we compared our data to a previous study [11] where PacBio Iso-Seq long read sequencing was performed on RNA from chicken embryonic hearts. The embryonic heart study yielded 1,566,465 reads that mapped to the Gal_gal_4 genome assembly. While the exact number of unique transcripts was not reported, 9221 novel isoforms were identified. We estimated the maximum number of unique transcripts that they could have acquired to be 31,081, which was calculated by adding their number for novel isoforms with the number of publicly annotated isoforms reported in their paper, 21,860 (16,743 from Ensembl and 5117 from RefSeq). Dividing their total possible number of unique transcripts by the number of reads they produced shows that, at most, only 2% of their reads were unique. While out of 482,325 mapped reads from our brain library, we found 55,315 (11.5%) unique transcripts. Thus the normalization method appears to have provided a transcriptome coverage efficiency of more than 5 times that of the previous study [11]. This means that for every SMRT cell used with the normalization method, 5 SMRT cells would be required without normalization to achieve the same amount of transcriptome coverage.

This transcriptome coverage efficiency calculation assumes that the previous study did not achieve full coverage of the transcriptome for their sample. While it is possible that they reached full coverage of their sample transcriptome, it seems unlikely since we found evidence for 44,898 transcripts from our chicken heart short read RNAseq data.

### Exploring the PacBio transcriptome of the chicken reference genome

#### Protein coding and noncoding RNA genes and transcripts

We used three methods to find evidence for protein coding potential: Blastx [22] with the Uniprot Uniref 90 protein database [23], the Coding Potential Calculator (CPC) software [24], and the Coding Potential Assessment Tool (CPAT) [25]. Combining the results from the three methods, we found 43,738 putative protein coding transcripts from 14,421 genes and 20,539 putative noncoding RNA transcripts from 17,178 genes (Table 1). Within the noncoding RNAs (ncRNAs), we found that 23 transcripts were shorter than 200 bp which means the rest were classified as long noncoding RNAs.

**Table 1:** Classification of biotypes for PacBio transcriptome

# of Transcripts	Biotype
43,738	Coding RNA
20,516	LncRNA
23	Short ncRNA
4735	NMD transcript
13,873	Antisense Exonic
2139	Antisense Intronic

We noticed a difference in the number of exons between coding and noncoding transcripts. There were 14,831 noncoding single exon transcripts (72.2%) and only 5533 protein coding single exon transcripts (12.7%) (Fig. 4e). Thus within this dataset single exon transcripts make up the majority of noncoding RNAs.

We classified the lncRNAs by positional relationship to the predicted protein coding transcripts. There were 12,999 long intergenic noncoding RNAs (lincRNAs), 2675 antisense lncRNA, and 4967 sense overlapping lncRNA.

#### Nonsense mediated decay products

Nonsense mediated decay (NMD) products are transcribed alternative splice variants from protein coding genes that are not translated into proteins [26–28]. NMD products have similar sequences to protein coding transcripts but typically have been spliced so that there is an early stop codon [27]. We used the NMD prediction rules outlined in NMD studies [27, 29–31], which state that a premature termination-translation codon occurring at least 50-55 base pairs upstream of a splice junction provides strong evidence for NMD. Using this criteria, we identified 4735 putative NMD transcript candidates within our PacBio data (Table 1).

Although Ensembl did not make NMD predictions for the Ensembl (release 83) chicken annotation, they did have predictions for the human and mouse annotations. We ran our NMD method on Ensembl (release 83) human and mouse annotations to compare our methods. Out of 13,401 Ensembl human NMD transcripts, 13,263 were predicted to be NMD using our method (99% agreement). From our NMD predictions, out of 79,901 Ensembl annotated human protein coding transcripts, only 909 were predicted to be NMD which equates to a false discovery rate (FDR) of 6.4%. Similarly for 5229 NMD transcripts in the mouse annotation, 5152 were predicted to be NMD using our method (99% agreement). Out of 50,706 Ensembl mouse protein coding transcripts, we predicted only 341 transcripts to be NMD which equates to an FDR of 6.2%.

When overlapping the PacBio NMD transcripts with the Ensembl annotation, we found that 4137 NMD transcripts overlapped with 2517 Ensembl genes. We looked at the ratio of the number of NMD to coding for both gene level and transcript level per chromosome and found that all chromosomes had a ratio at gene level between 0.16 and 0.29 (Fig. 4f). We ran the same analysis on the Ensembl (release 83) human and mouse annotations and found similar ratios ranging from 0.12-0.5 and 0.03-0.26 respectively.

#### Identification and classification of antisense transcripts

In the chicken PacBio transcriptome, there are 13,873 transcripts that are exonic antisense overlapping to at least one transcript and 6446 genes that are antisense to at least one gene. We looked at the numbers of coding and noncoding transcripts with respect to these antisense transcripts and found that there were 7107 transcripts involved in a protein coding to noncoding antisense overlap, 4765 transcripts involved in protein coding to protein coding antisense overlap and 2001 transcripts for noncoding to noncoding antisense overlaps (Table 1). When converting these to gene antisense overlap pairs we found 1571 protein to noncoding gene pairs, 1329 protein coding to protein coding gene pairs and 1036 noncoding to noncoding gene pairs.

Looking at intronic antisense overlap, where transcripts have at least one exon that overlaps with the intron of an antisense transcript, we found 2139 transcripts and 1115 genes with at least one antisense intronic overlap (Table 1). When considering coding predictions we found 354 protein coding to noncoding, 298 protein coding to protein coding, and 140 noncoding to noncoding gene pairs.

#### Transcriptional complexity in the chicken genome

Due to the large number of unique transcripts that were identified, we were able to make a general assessment of transcriptional complexity in the chicken genome. We looked at the ratio of transcripts to genes, retained introns, skipped exons, alternative exon starts/ends, alternative TSS and TTS, and single exon transcripts.

**Fig. 5:**
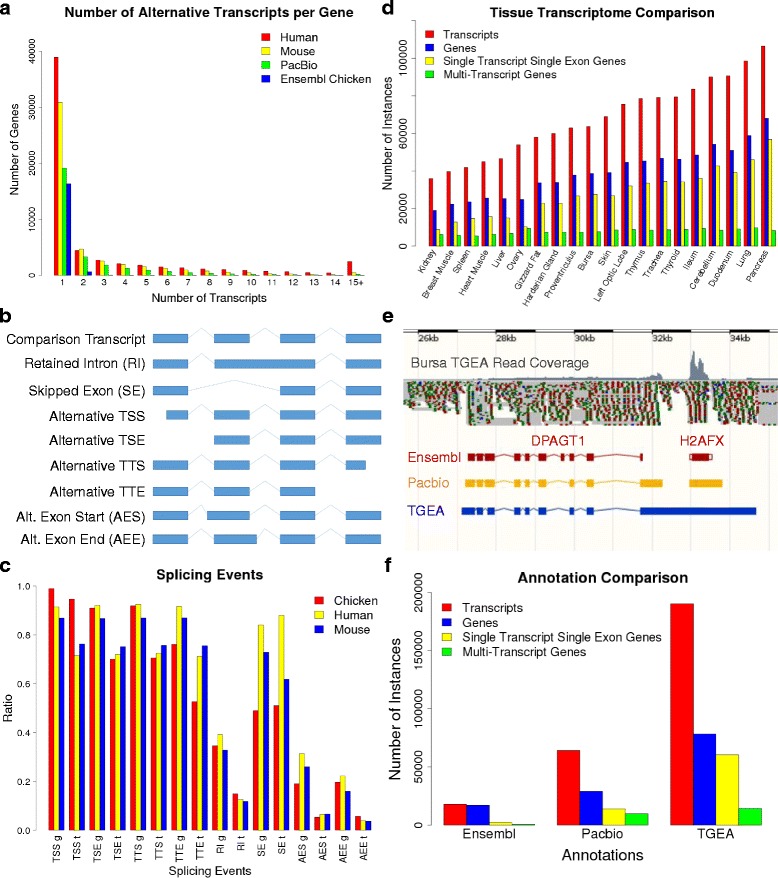
Alternative splicing. **a** Comparison of number of alternative transcripts per gene between Ensembl annotations and PacBio transcriptome. **b** Classifications for alternative transcripts. **c** Comparison of rate of occurrence for the different classes of alternative transcripts between Ensembl human, mouse, and the chicken PacBio Transcriptome. Abbreviations for x-axis labels explained in Fig. 5b. **d** Comparison between STSE and MT genes for TGEA transcriptome. **e** Example of overhang event. **f** Comparison of the number genes and trranscripts for Ensembl, PacBio and TGEA

We found a ratio of 2.22 for transcripts to genes. This low number is due to the abundance (19,120 genes of which 13,265 are lncRNA genes) of genes with single transcripts many of which are single exon genes. If these single transcript genes are removed then the ratio increases to 4.56 transcripts to genes (Fig. 5a). These numbers are likely an underestimation as we have only characterised two, albeit transcriptionally complex, tissue types and some lowly expressed transcripts may have been missed in our brain and embryo libraries.

For assessing alternative TSS we only used the transcript sequences from the embryo library since this library had 5′-cap selection and therefore should have intact 5′-sequences. We removed all genes with only one representative transcript since these would by default have only one TSS. There were 2037 genes that matched these criteria and only 73 had only one TSS. Thus 96.4% of these genes had multiple TSS. The high rate of multiple TSS genes is presumed to be a combination of transcription factor binding wobble and alternative transcription start exons (TSE). If we ignore TSS caused by wobble and only look at TSE, 594 genes have a single TSE which means 70.8% of these genes have multiple starting exons.

For TTS we use both brain and embryo transcript sequences since both libraries had been selected for poly-A tails. Again all single transcript genes were removed which resulted in 9893 genes. Only 801 genes had a single TTS which means that 91.9% had multiple TTS. However, if we look at alternative transcription termination exons (TTE), we find that 2365 genes have a single TTE which means 76.1% have multiple TTE.

We also looked at occurrences of retained introns and skipped exons within both brain and embryo transcripts using only multi-transcript genes. We define retained introns as exons which overlap an entire intron (Fig. 5b) from another transcript. There are 3429 multi-transcript genes which have retained introns which equates to a rate of 34.7%. We define skipped exons as exons which are completely overlapped by an intron in another transcript (Fig. 5c). There were 4939 genes with at least one occurrence of skipped exons which equates to a rate of 49.9%.

We looked at alternative exon start (AES) and ends (AEE). For this set we used both brain and embryo transcripts but only assessed internal exons so that we did not include TSE and TTE. We also excluded retained intron exons from this set. There were 8006 genes with no AES which equates to a rate of 19.1%. There were 7952 genes with no AEE which equates to a rate of 19.6%. So the rates of AES and AEE are quite low as compared to other alternative splicing events.

We were interested to see if there were any alternative splicing differences between protein coding and lncRNA genes. Out of 14,421 protein coding genes, 6597 had only one transcript which gives a multi-transcript rate of 54.3%. Out of 17,178 lncRNA genes, 15,162 had only one transcript which gives a multi-transcript rate of 11.7%. Thus lncRNA genes are much less likely to contain alternative transcripts. We also noticed that lncRNA transcripts were much more likely to have only one exon. Out of 20,539 lncRNA transcripts, 14,831 contained only a single exon. Whereas, out of 43,738 protein coding transcripts, there were only 5533 single exon transcripts. LncRNA transcripts had a rate of 72.2% for single exon transcripts as compared to 12.7% for protein coding transcripts. When adjusting the multi-transcript rate for only multiple exon genes, there is a rate of 67.0 and 37.0% for coding and lncRNA genes respectively. Thus even after accounting for the high number of lncRNA single exon genes, coding genes are more likely to have alternative transcription.

#### Comparison of transcriptome assemblies derived from short and long read RNA sequencing data

We created a tissue gene expression atlas (TGEA) derived from the assembly of short read RNAseq data of 20 tissue types from J-line layer chickens (Table 2) to compare and independently validate the PacBio transcriptome. We merged the identified transcripts from each short read RNAseq tissue dataset into a single transcriptome annotation to create the TGEA.

**Table 2:** Number of transcripts and genes by tissue type for TGEA transcriptome

Tissue	# of Transcripts	# of Genes
Kidney	35,867	18,916
Breast Muscle	39,649	22,357
Spleen	41,831	23,546
Heart Muscle	44,898	25,520
Liver	46,523	25,253
Ovary	53,933	24,787
Gizzard Fat	57,922	33,670
Harderian Gland	59,873	33,791
Proventriculus	62,954	37,824
Bursa	63,644	38,673
Skin	68,982	39,211
Left Optic Lobe	75,457	44,567
Thymus	78,491	45,312
Trachea	79,103	46,730
Thyroid	79,440	46,285
Ileum	83,541	48,446
Cerebellum	90,088	54,212
Duodenum	90,665	50,902
Lung	98,514	58,762
Pancreas	106,430	68,006

The TGEA transcriptome predicts 78,351 genes with 190,474 transcripts. Thus the TGEA has 2.7 times the number of genes and 2.96 times the number of transcripts as compared to the PacBio transcriptome. While this difference is most likely explained by the inclusion of many more tissue types in the TGEA as compared to the PacBio transcriptome, there are also some fundamental differences in the proportion of multiple transcript and single transcript single exon (STSE) genes (Fig. 5d). When only comparing multiple transcript genes, the PacBio transcriptome has 9893 genes while the TGEA transcriptome has 14,220 genes. However, for STSE genes, PacBio has 13,824 genes while TGEA has 60,576 genes. Thus the ratio of STSE genes to multiple transcript genes is 1.40 in PacBio and 4.26 in TGEA. Of the total STSE genes in the PacBio transcriptome, 12,603 are classified as lncRNA.

We looked at genomic overlap between PacBio and TGEA transcripts to estimate the transcript coverage for each dataset. There were 9368 PacBio transcripts which had no overlap with TGEA transcripts. However, when we guide the transcript assembly for the TGEA dataset using the PacBio transcripts, we find that only 18 PacBio transcripts have no coverage. This indicates that despite the high depth of sequencing and wide tissue coverage of the TGEA dataset, a large number of transcripts were not predicted even though there were data to support their existence. This may be a result of the difficulty in differentiating transcriptional noise from true transcripts. Thus the PacBio transcripts missing in the TGEA transcriptome were difficult to distinguish from noise using short read data.

There were 108,651 transcripts from 15,633 genes in the TGEA which overlapped the PacBio transcriptome. So 43% of the TGEA transcripts and 80% of the TGEA genes are not covered by the PacBio transcriptome. However, of these TGEA models with no PacBio transcript overlap, 76.6% of the transcripts and 91.5% of the genes are from single transcript single exon genes. While these may represent true transcripts and genes, it is difficult to be sure that these are not the result of transcriptional noise using only short read evidence.

We noticed during manual inspection of the PacBio and TGEA overlaps that some transcript models in the TGEA transcriptome seemed to be a merging of two adjacent genes in the PacBio transcriptome. We call this event an “overhang gene” (Fig. 5e). To investigate the abundance of these events, we searched for all TGEA transcripts which overlapped two PacBio genes. We identified 2515 overhang events where an upstream and downstream PacBio gene is represented as one merged gene in the TGEA transcript model. Of these, 208 events occur where the downstream gene model has a confirmed start site due to the presence of transcript models from the embryo data. Out of these, 79 overhang events have external support from Ensembl (release 83) chicken annotation showing that each gene is a separate well annotated gene. To understand the more general problem of gene merging we looked at all gene merging events where one TGEA transcript merged two or more PacBio genes. We identified 4254 merged gene events involving 10,991 PacBio genes.

#### Comparison of the PacBio transcriptome with public annotation

Ensembl and NCBI are the two major sources of public annotation for the chicken genome. Since the NCBI chicken annotation contains far fewer transcripts and genes (6352 and 6027 respectively) as compared to the Ensembl chicken annotation (release 83) and 96.8% of the transcripts in NCBI are also contained within Ensembl, we chose to focus our analyses on the Ensembl annotation. The Ensembl (release 83) chicken annotation contains 17,108 genes with 17,954 transcripts. There are 15,508 genes annotated as protein coding, 42 predicted as pseudogenes, 150 ambiguous RNA, and the rest are an assortment of short noncoding RNA. There are no annotated lncRNA. It has a ratio of 1.05 transcripts per gene model with only 745 multiple transcript genes. For these multiple transcript genes, the ratio of transcripts per gene is 2.14. The PacBio transcriptome has a ratio of 2.22 transcripts per gene when including the entire gene set and 4.56 transcripts per gene for multiple transcript genes (Fig. 5f). The Ensembl (release 83) chicken annotation contains 969 antisense genes as compared to 6446 antisense genes for the PacBio transcriptome. These are genes that overlap at least one gene on the opposite strand.

Out of the 64,277 PacBio transcripts, 21,887 had no overlap with Ensembl transcript models and are thus considered to be novel. Of these, 7414 transcripts had no sense exonic overlap with any Ensembl predicted transcript but were either antisense (exonic or intronic) or had a sense intronic overlap. These transcripts could be further classified based on their coding potential so that 5049 were noncoding and 2365 were protein coding (Table 3). The remaining transcripts were located in intergenic regions. Of these, 11,880 were predicted to be noncoding while there were 2593 intergenic coding transcripts (Table 3).

**Table 3:** Classification of coding and noncoding transcripts by gene overlap

# of Transcripts	Coding class	Exonic antisense	Intronic antisense	Intronic sense
1634	ncRNA	Yes	No	No
1262	ncRNA	No	Yes	No
2047	ncRNA	No	No	Yes
32	ncRNA	Yes	No	Yes
74	ncRNA	No	Yes	Yes
11,880	ncRNA	No	No	No
1478	coding RNA	Yes	No	No
200	coding RNA	No	Yes	No
575	coding RNA	No	No	Yes
55	coding RNA	Yes	No	Yes
57	coding RNA	No	Yes	Yes
2593	coding RNA	No	No	No

#### Comparative genomics and phylogenomic profiles of chicken PacBio transcripts

To understand the conservation of these sequences across birds and other vertebrate species, we mapped the transcript sequences (using GMAP with default parameters [16]) onto the genomes of several avian species as well as representatives from other vertebrate classes (Table 4). The avian species with the best genome assemblies for each phylogenetic grouping were selected for this analysis. We used this selection criteria so as to avoid mapping biases from low quality genome assemblies. These mappings do not directly relate to orthologs but rather provide a general indication of transcript sequence conservation between species.

**Fig. 6:**
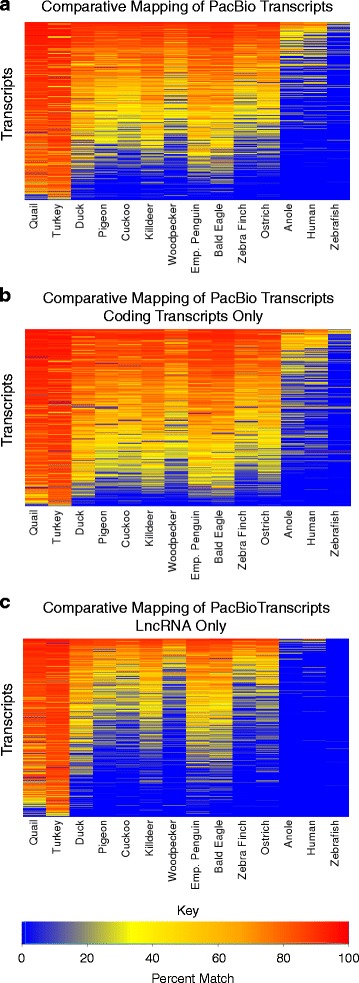
Comparative mapping of PacBio transcripts. **a** Heatmap of PacBio transcripts mapped onto other species’ genome assemblies. **b** Only the coding transcripts. **c** Only the lncRNA transcripts

Only 0.2% of the total chicken PacBio transcripts did not map to any non-chicken species. While 8.8% of the chicken PacBio transcripts mapped to all species spanning 300Mys. Of these, 98.8% were predicted to be protein coding while 1.2% were predicted to be lncRNA. Of the lncRNA that mapped to all species, 52.3% were predicted to be intergenic. When focusing only on avian species, we see that 61.8% of the chicken PacBio transcripts map to all avian species. From these transcripts which map to all avian species, we see 82.8% predicted as protein coding and 17.2% predicted to be lncRNA (percentages given with respect to the total number of transcripts which map to all avian species included in this analysis). Out of the lncRNA transcripts that mapped to all avian species, 47.1% are classified as lincRNA. We produced heat maps to display this analyses with a colour scale indicating the quality percent of mapping for each transcript (Fig. 6 a-c). The quality percent is defined by the number of matching nucleotides divided by the total length of the transcript when aligning the chicken PacBio transcripts with their projected sequence when mapped to other species.

## Discussion

### Noncoding transcripts

#### Long noncoding RNA

In the Ensembl (release 83) annotation, there are 24,149 lncRNA transcripts predicted in human and 8391 predicted in mouse. Thus our 20,516 predicted lncRNA transcripts are similar in number to that found in the human annotation, which has the highest number of annotated lncRNAs of any Ensembl annotated vertebrate genome. While the mouse annotation usually benefits from homology based predictions from humans, the lack of conservation for lncRNA sequences has made homology methods mostly ineffective. Previous studies have shown that a large proportion of the human lncRNA are primate specific [32], which would explain the comparatively low number of identified lncRNA in mouse. The similar numbers of identified lncRNA in the Ensembl human annotation and the PacBio chicken annotation suggests that lncRNA are extremely underrepresented in the annotations of mouse and other species.

The Ensembl annotated lncRNAs are classified into three main categories: lincRNA, sense overlapping lncRNA, and antisense lncRNA. However, it is important to note that there are no biotype designations for sense exonic overlapping lncRNA in the Ensembl annotation. The sense overlapping class is comprised of two Gencode defined biotypes termed sense_overlapping and sense_intronic. Sense_overlapping refers to lncRNA transcripts that have a protein coding gene within their introns. Sense_intronic refers to lncRNA transcripts that occur within the intron of a protein coding gene. Neither of these correspond to any exonic overlap, thus they are both sense intronic overlapping lncRNA. There is, however, a biotype classification labelled “processed_transcript” which is defined as a transcript with no open reading frame. There are transcript models within this group which meet the criteria for sense exonic lncRNA, however, due to the lack of evidence to support these models it is unclear how many represent true sense exonic lncRNA. Thus there are three sub-classes for lncRNA within the Ensembl annotation with a loosely defined 4^th^ class which contains sense exonic lncRNA but not at an annotation level that can be used with high confidence. This means that the proportion of sense exonic lncRNA in human and mouse is unknown.

**Fig. 7:**
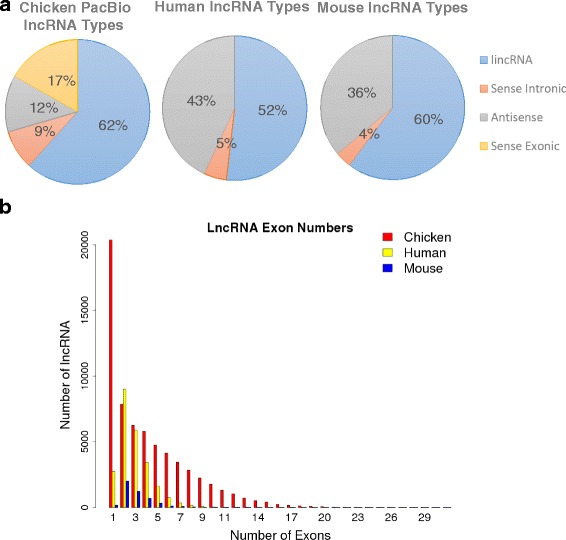
Characterization of lncRNA. **a** Proportions of each class of lncRNA for chicken PacBio, Ensembl human, and Ensembl mouse annotations. **b** Proportion of exon numbers for lncRNA transcripts for chicken PacBio, Ensembl human, and Ensembl mouse annotations

For both the human and mouse annotation, lincRNA make up roughly half of the total, while sense intronic lncRNA represent less than 10% of the total (Fig. 7a). Thus proportions of these classes seem to be well conserved within mammals. However, the relative proportions of the lncRNA sub-classes in the PacBio chicken annotation are very different. This difference seems to be due in large part to the inclusion of sense exonic overlapping lncRNA which make up 17% of PacBio chicken lncRNA transcripts (Fig. 7a). This difference could represent real biological differences between mammalian and avian genomes such that antisense lncRNA are more common in mammals while sense overlapping lncRNA are more common in birds. However, when we used our sense exonic overlap prediction tool on the Ensembl human and mouse processed_transcript models, we found 24,385 and 11,901 sense exonic lncRNA transcripts respectively. If these numbers are included in the proportion of lncRNA types then they would equate to 48 and 57% respectively. This would indicate that sense exonic lncRNA are actually the most abundant type of lncRNA. However, due to the dearth of evidence for these models, it is difficult to say whether this reflects reality. The proportions of lncRNA sub-classes within the PacBio chicken annotation may provide an estimate for the rate of occurrence of sense exonic lncRNA in human and mouse as well as other vertebrate species.

Another startling contrast between the human/mouse Ensembl (release 83) annotations and the PacBio chicken annotation is the proportion of the number of exons for lncRNA transcripts. In the human/mouse Ensembl annotations, two exon lncRNA transcripts are the most commonly occurring (Fig. 7b). However, the PacBio chicken transcriptome show that single exon lncRNA transcripts are by far the most abundant. While this difference could be due to real biological differences between birds and mammals, no conclusions can be made because many of the lncRNA prediction methods for the human and mouse annotations removed single exon lncRNA models [33]. The practice of removing single exon lncRNA models is useful when dealing with models that are assembled from short read data since it is difficult to ascertain whether these models are truly single exon transcripts or the result of transcriptional noise. However, this puts a strong bias against the prediction of single exon lncRNA transcripts which has likely resulted in the underrepresentation of these transcripts. Thus the proportion of single exon lncRNA transcripts in the PacBio chicken annotation may indicate that these are also the largest group of lncRNA in other vertebrate species. If this is true, then a large portion of lncRNA have not been identified due to the practice of filtering out single exon lncRNA models.

#### Non-sense mediated decay transcripts

In comparison to the proportion of NMD products in human and mouse, our NMD predictions for chicken appear to be similar. Our predictions for NMD in chicken also show a more uniform ratio of NMD to coding transcripts across the chromosomes. Since 2517 Ensembl genes have NMD overlap, it appears that NMD may play a large role in protein expression regulation within the chicken. Considering the important biological implications of NMD products [34], the lack of annotated NMD transcripts in the public chicken annotation could have concealed important gene expression information in previous studies.

### Antisense genes

The most common pairing for both exonic and intronic antisense genes is that of a protein coding gene with a noncoding gene which is supported by reports in mammals [35, 36]. The predominance of the coding to noncoding pairs suggests that there may be some regulatory relationship between the coding and noncoding genes in each pairing. While the mechanism of regulation is still mostly unknown, it has been proposed that one way in which an antisense gene can regulate a sense gene is by inhibiting transcription of the sense gene through transcriptional collision [37]. Thus the protein coding genes within these antisense pairings may be down regulated by the transcription of their noncoding antisense partners. In these situations, it is the action of transcription that is functional as opposed to the transcriptional product. Thus the sequence of the antisense partner is essentially meaningless and almost completely free of selection pressure (aside from exonic overlapping regions). This would explain why lncRNA sequence conservation is so low as compared to protein coding genes.

The abundance ranking of antisense pairs from coding to noncoding, coding to coding, and then noncoding to noncoding has also been found in mammals [35]. The consistency of this ranking order within this study and within mammalian studies stands out as a peculiar coincidence. It suggests that coding to noncoding antisense regulation is a widely adopted and significant form of regulation within vertebrates. The coding to coding pairs may be a relic of ancient genomes where genomic compactness offered some selective advantage. However, it is perplexing as to why noncoding to noncoding pairs would be the least abundant. Due to the lack of sequence conservation for lncRNA, many believe that the majority of lncRNA lack function and refer to them as transcriptional noise. If they are truly non-functional, then their transcription near functional genes would likely have negative effects for several reasons. For instance, competition for access to the region by transcription factors. If lncRNA are predominantly non-functional, it is more likely for them to occur near each other and not near useful genes. Thus lncRNA genes should make up the most abundant antisense pairs. The growing evidence that noncoding to noncoding pairs are the least abundant suggests that the majority of lncRNA are functional and their sequences are functionally important as well.

The prevalence of exonic pairs over intronic pairs offers another unintuitive result. Since intronic pairs have less sequence dependency between the two genes, it seems more probable for these pairings to arise. Yet there are less than a sixth of the amount of intronic gene pairs as compared to exonic. This large discrepancy suggests that there is some functional reason for why antisense exonic pairs dominate. It may be that the exonic sequence overlap allows RNA binding between the antisense products which could be used for up or down regulation. If this were the case, then perhaps the majority of antisense pairings represent a regulation relationship between the antisense genes. This would make sense from transcriptional collision alone but the RNA binding theory may add another level of regulation tuning.

### Alternative transcription events

Multiple transcript genes within the PacBio chicken transcriptome display a high level of complexity with regard to transcription initiation and termination which is similar to that found in mammalian annotations [38]. When comparing the different alternative transcription events (Fig. 5b), the most dominant events are alternative TSS and TTS. This result matches the human and mouse annotations (Fig. 5b). It is possible that the TSS is a major factor in determining the splicing of the transcript such that each alternative transcript is somewhat defined by the TSS. This would mean that the polymerase binding site defines the alternative transcript which would be a mechanism for regulating alternative transcription.

Skipped exon events are the most dominant alternative splicing event with 49.0% of the PacBio genes having at least one occurrence and a rate of 51.0% for PacBio transcripts. While skipped exon events are the most dominant in both the human and mouse annotations, they occur at higher rates, 84.1 and 72.8% of genes, respectively. This may indicate that they play a lesser role comparatively in the chicken genome. The lower relative rate of occurrence for skipped exons in the chicken transcriptome may also contribute to the density of the genome, since skipped exons are related to greater overall intronic regions as they are effectively introns for other transcripts.

For retained introns, alternate exon starts, and alternate exons ends, there is a significant difference between the rate of occurrence when calculating per gene versus per transcript, with each event having a higher per gene rate than per transcript rate. This means that they tend to be spread out among genes but with fewer occurring within each gene. This contrasts the rates for skipped exons where there is actually a higher rate per transcript than per genes in the PacBio transcriptome. This suggest that these events may be related to a type of RNA product which does not benefit from a variety of these events, such as NMD products, where introducing an early stop codon is all that is needed.

### Long read versus short read RNA sequencing data

If we consider the TGEA as a representation of an upper limit for transcribed loci discovery, then the difference in ratios between TGEA and PacBio for STSE to multiple transcript genes suggests that the majority of unannotated transcribed regions are STSE genes. However, another explanation is that many of these novel STSE genes in the TGEA transcriptome are a result of RNAseq noise. Since STSE genes have no splice junctions, there is no other supporting evidence for the existence of these genes except for read coverage. Since read coverage for a specific locus may be influenced by sequence similarity to another locus or errors in the genome assembly, it is possible that the supporting reads belong to another locus. It is also possible that a STSE gene is actually an exon from another gene, but due to issues with low read coverage not linking the exon to the rest of the gene, the model was predicted incorrectly. Thus it is difficult to say how many of the TGEA transcipts/genes are accurate.

On the other end, the 9368 PacBio transcripts with no overlap from the TGEA indicates that there may be many transcripts which go undetected with short read sequencing. This under-prediction can be the result of genes with low expression levels or genes with sequence similarity to other loci (such as paralogs).

The relatively large number of gene merging events (4254) in the TGEA transcriptome indicate a clear issue with transcriptomes assembled from short read RNAseq data. Each gene merge event represents an incorrect transcript model that would be misidentified using standard annotation pipelines that rely on open reading frames and transcript length. While investigating gene merge events, we noticed that the transcript assembly errors seemed to be a result of short read noise. This noise is manifested as a low coverage of reads over intronic and intergenic areas. These noisy reads can bridge between transcripts thus resulting in merged gene models. This noise also makes it difficult to detect retained introns. Due to issues with noise, most assemblers use some method of thresholding to decipher when intronic reads are noise or real. However, filtering out noise reads from real reads is non-trivial and relies on low variance of read coverage over the transcript, which is rarely the case. Therefore, while the TGEA dataset can provide a rough estimate of transcribed loci, it is not recommended for identifying full length transcript sequences.

### Comparing the PacBio transcriptome to the Ensembl annotation

The large difference in the number of anti-sense genes between Ensembl and PacBio is partly explained by the greater number of genes in the PacBio chicken transcriptome. However, it is also indicative of the limitations of the short read RNAseq data that was used for the Ensembl chicken annotation [5, 39]. Much of these data were generated using unstranded library preparation protocols which made it impossible to resolve anti-sense transcripts. Without stranded RNAseq data, anti-sense transcripts can look like extensions of the sense transcripts or can be filtered due to their non-conformance with the dominant transcript model. As a result, these models may have been omitted or represented incorrectly.

Due to the lack of lncRNA models in the Ensembl annotation, the large number of novel ncRNA predicted by PacBio sequencing is somewhat expected. The number of novel intergenic protein coding transcripts, however, was higher than we expected so we investigated the possible reasons for their absence in the Ensembl annotation. There were 634 transcripts which had no hits against the Uniref 90 database. This subset represents transcripts with no or low sequence similarity to known proteins. Ensembl may have discarded these in their pipelines since they would be difficult to confirm as protein coding. These also represent possible avian specific proteins. There were 891 transcripts which were antisense (exonic or intronic) to a transcript either in the PacBio annotation or the TGEA. Similar to the transcripts that were antisense to Ensembl transcripts, these transcripts represent complex transcribed loci where short read data may not provide enough information to resolve the overlapping transcripts. There were 719 transcripts which did not have matching transcript models in the TGEA. Thus these transcripts could not even be assembled with short read data. This is mostly likely due to low and/or variable coverage. There were 967 transcripts left after removing the no hit, antisense, and no TGEA sense overlap transcripts. Therefore the majority of these previously unannotated transcripts can be explained by the limitations of short read RNA sequencing.

#### Comparative genomics provides functional support for PacBio transcript predictions

The mapping of over 99.8% of PacBio chicken transcripts to other genome assemblies provides support for the PacBio models and sequence conservation also predicts functional constraints on these transcripts. While there is some variability of genome assembly quality among the avian species, there is a clear trend for species that are evolutionarily closer to chicken to have more matching transcript sequences. The galliformes show a high proportion of similar transcripts (Fig. 6a), while there is a dropping off of the number of mapped transcripts for species at a further phylogenetic distance away from chicken. For the non-avian species, relatively few transcript mappings show similarity.

Comparing the coding and lncRNA transcripts, we see that there is a very different trend for sequence conservation with coding transcripts being relatively well conserved across the avian species (Fig. 6b) while a more noticeable drop off occurs with the lncRNA transcripts (Fig. 6c). This complements previous observations that lncRNA have low conservation as compared to protein coding genes [40] and homology approaches have limited effectiveness beyond closely related species.

## Conclusions

We identified a large number of events where transcriptional complexity make it difficult or impossible to attain the true transcript sequences from short read data. As the current public annotation of the chicken genome by Ensembl has relied upon incomplete cDNA sequences (ESTs) and short read RNA-seq data, the complexity of the chicken transcriptome is currently underrepresented. The current underrepresentation of transcriptional complexity with respect to the number of alternative transcripts can have consequences for analyses that rely on these models. Important transcriptional events can be missed or misrepresented thus obscuring underlying biological processes. Using PacBio sequencing to create a high quality transcriptome annotation can correct these issues that are common in many of the public annotations. More advanced analytical tools can be developed to take advantage of the long read transcriptome by using information which could identify problematic areas in short read data during transcript quantification experiments. These areas include multi-mapping loci, repeat regions and ambiguous splice junctions.

Long read transcript models also improve functional annotation since many annotation pipelines must assume that the supplied transcript sequences represent real splicing and correct reading frames. The ability to disambiguate overlapping transcripts or genes sheds light on transcriptome complexity that was previously unannotated in chicken. The PacBio chicken transcriptome suggests a level of transcriptional complexity that is more consistent with expectations based on the well-characterised human genome.

## Methods

### Pacific Biosciences Iso-Seq long read sequencing

For the brain library, brain tissue was collected from an adult J-Line chicken (brown leghorn) bred at the Edinburgh Poultry Research Centreand the extracted RNA sample was sent to GATC Biotech (Konstanz, Germany) for library preparation and sequencing. The total RNA sample was examined using capillary electrophoresis with a Shimadzu MultiNA microchip electrophoresis system (Shimadzu Corporation, Kyoto, Japan). Poly(A) + RNA was selected using an oligo(dT)-linker primer and cDNA was produced using M-MLV H- reverse transcriptase. The cDNA was amplified using PCR with 16 cycles. Normalization was performed by denaturing and reassociating the cDNA. Double stranded cDNA were removed using a hydroxylapatite column. The remaining cDNA were then amplified using PCR with 8 cycles. The cDNA were then size selected for 1 kb and 2 kb lengths using Ampure beads (Agencourt BioSciences Corporation, Beverly, Massachusetts). For the 1 kb cDNA, 11 SMRT cells were used. For the 2 kb cDNA, 14 SMRT cells were used.

For the embryo library, an embryo was collected at Hamburger-Hamilton stage 26 from an ISA Brown chicken bred at the Edinburgh Poultry Research Centre and RNA was extracted. The RNA sample was sent to GATC Biotech for library preparation and sequencing. The total RNA sample was examined using capillary electrophoresis with a Shimadzu MultiNA microchip electrophoresis system. Poly(A) + RNA was selected and treated with Terminator exonuclease (+TEx). The 5′CAP structures were removed using tobacco acid pyrophosphatase (TAP). Then an RNA adapter was ligated to the 5′-monophosphate of the RNA. The cDNA was synthesized using an oligo(dT)-adapter primer and M-MLV H- reverse transcriptase. The cDNA was amplied using PCR with 13 cycles. Normalization was performed by denaturing and reassociating the cDNA. Double stranded cDNA were removed using a hydroxylapatite column. The remaining cDNA were then amplified using PCR with 8 cycles. The resulting cDNA was purified with the Agencourt AMPure XP kit. The cDNA were then size selected for 0.8 kb and 2 kb lengths using Ampure beads. For the 0.8 kb cDNA, 16 SMRT cells were used. For the 2 kb cDNA, 17 SMRT cells were used.

### Long read transcriptome processing

Raw data was processed into error corrected reads of insert (ROI’s) using the PacBio SMRT Analysis Package with default parameters. The ROI’s were then processed using the Iso-Seq Tofu pipeline [15]. We used the Classify module with default parameters to remove adapter sequences, poly-A tails, artificial concatemers, and 3′ truncated transcript sequences which resulted in our set of FLNC transcripts. For an additional level of error correction we ran PacBio ICE software without the Quiver step on the FLNC transcripts [15]. The ICE software clusters transcripts by alignment using BLASR and then error corrects using the alignments. This results in a higher quality set of transcript sequences and the removal of redundant data. Due to the computation time restraints we ran ICE independently on each size selection from the brain.

The resulting sequences were then mapped to the Galgal 4 reference genome assembly using GMAP [16] using default parameters. The GMAP result bam files are then processed using the Iso-Seq Tofu Collapse module (in the Iso-Seq pipeline) which merges transcripts based on genomic coordinates. There are two methods of doing this which are explained in the Results section.

For each transcript we collected the quality scores of the ICE cluster sequences contributing to that transcript model. We estimated quality score by aligning the pre-mapped sequence to the post-mapped sequence using MUSCLE [41] and counting the number of mismatches and gaps. We then took the longest supporting cluster for each transcript and removed the transcript from our working list if the quality percentage of the longest supporting cluster was less than 90%.

ROI quality scores were calculated with a similar method. Adapter sequences were aligned to the ROI sequences using MUSCLE and the quality score was calculated by counting the number of mismatches.

The collapsed transcripts from the brain library and the embryo library were then merged using in-house python scripts to create a PacBio transcriptome annotation.

### Illumina RNA sequencing

RNA samples from 20 tissue types were collected from 9 16/17 weeks old female J-Line chickens bred at the Edinburgh Poultry Research Centre . The samples from the 9 individuals were pooled for each tissue type and sequenced by Edinburgh Genomics. The Illumina Total RNA Stranded kit was used to generate stranded cDNA fragments. In this stranded RNAseq method, random primers are used for reverse transcription to create a complementary strand to the original RNA template. Deoxyuridine Triphosphate (dUTP) is then incorporated into the original template [42]. Adapters are attached to both ends of the double strand and then the original template is degraded. The adapters provide strand information based on their orientation in the read. The cDNA fragments were then sequenced to produce paired end reads with an average length of 101 base pairs. The average size of cDNA fragments was 190 bases.

### Short read transcriptome assembly

Edinburgh Genomics generated 8 fastq file pairs for each tissue. Each tissue had an average of 120,563,969 reads between all 8 fastq files. We checked the quality of data using Fastqc. We then mapped the reads to the Galgal 4 genome assembly using Tophat2 version 2.0.14 with Bowtie2 version 2.2.5. For this we used the parameters to define the inner insert size for each library and the strand orientation (--library-type fr-firststrand). We then ran Cufflinks version 2.2.1 to assemble transcripts using default parameters on each library (8 libraries per tissue). No annotations were provided at this step for guiding. We then merged the transcript models from each library using Cuffmerge. This final merged annotation was designated as our J-line derived annotation.

We also ran Cufflinks using force guided on the Ensembl (release 83) annotation, the PacBio annotation, and the J-Line derived annotation. This was performed to acquire FPKM estimates for each transcript model in each annotation set so that we could generate our expression atlases.

### Other bioinformatics analyses

#### Prediction of coding and noncoding transcripts

To classify the PacBio transcripts as either protein coding or noncoding we used the criteria that transcripts without evidence for protein coding potential were labelled as noncoding RNA and transcripts with evidence were labelled as putative protein coding. We used three methods to find evidence for protein coding potential. The first method consisted of using Blastx [22] to find hits between the PacBio transcripts and the Uniprot Uniref 90 protein database [23]. The second method consisted of using the Coding Potential Calculator (CPC) software [24]. CPC uses six different metrics to determine the coding potential of a transcript. These include using open reading frames (ORFs) and searching for Uniprot protein hits. The third method consisted of using the Coding Potential Assessment Tool (CPAT) [25]. CPAT uses ORF’s and hexamer usage bias to produce protein coding probabilities. We used the recommended cut off of 0.3 for CPAT, designating any scores below this as noncoding.

#### Prediction of nonsense mediated decay products

Transcripts which were first identified as protein coding using our previously defined methodology were used for our NMD prediction. We investigated potential NMD products by identifying coding sequence regions within the PacBio transcript models. Coding sequence regions were identified by first converting the RNA sequences to peptide sequences in all three frames (single stranded data). The longest three ORF’s were matched to the Chicken protein sequences from Uniprot using Blastp [22]. The ORF’s with the highest match to a chicken protein were used as the representative or if no matches were found, the longest ORF was used. If the representative ORF had a stop codon that was more than 50-bp upstream of the final splice junction, it was labelled as an NMD candidate [31].

## Additional file

Additional file 1: PacBio sequencing read lengths (pdf format) (a) Read lengths for chicken brain 1-kb size selection. (b) Read lengths for chicken brain 2-kb size selection. (c) Read lengths for chicken embryo 0.8-kb size selection. (d) Read lengths for chicken embryo 2-kb size selection. (PDF 184 kb)

## Abbreviations

AEE: Alternative exon end
AES: Alternative exon start
ASV: Alternative splice variant
CPAT: Coding Potential Assessment Tool
CPC: Coding Potential Calculator
ECC: Exon Cascade Collapse
ICE: Iterative clustering for error correction
lincRNA: Long intergenic noncoding RNA
lncRNA: Long noncoding RNA
ncRNA: Noncoding RNA
NMD: Nonsense mediated decay
PacBio: Pacfic Biosciences
STSE: Single transcript single exon
TGEA: Tissue gene expression atlas
TSE: Transcription start exon
TSS: Transcription start site
TSSC: Transcription Start Site Collapse
TTE: Transcription termination exon
TTS: Transcription termination site
