# Supplementary material for: Normalized long read RNA sequencing in chicken reveals transcriptome complexity similar to human
Source: BMC Genomics. 2017 Apr 24;18:323. doi: 10.1186/s12864-017-3691-9 (PMC5404281; doi:10.1186/s12864-017-3691-9)
Supplement: Additional file 1: — PacBio sequencing read lengths (pdf format) (a) Read lengths for chicken brain 1-kb size selection. (b) Read lengths for chicken brain 2-kb size selection. (c) Read lengths for chicken embryo 0.8-kb size selection. (d) Read lengths for chicken embryo 2-kb size selection. (PDF 184 kb) [file 12864_2017_3691_MOESM1_ESM.pdf]

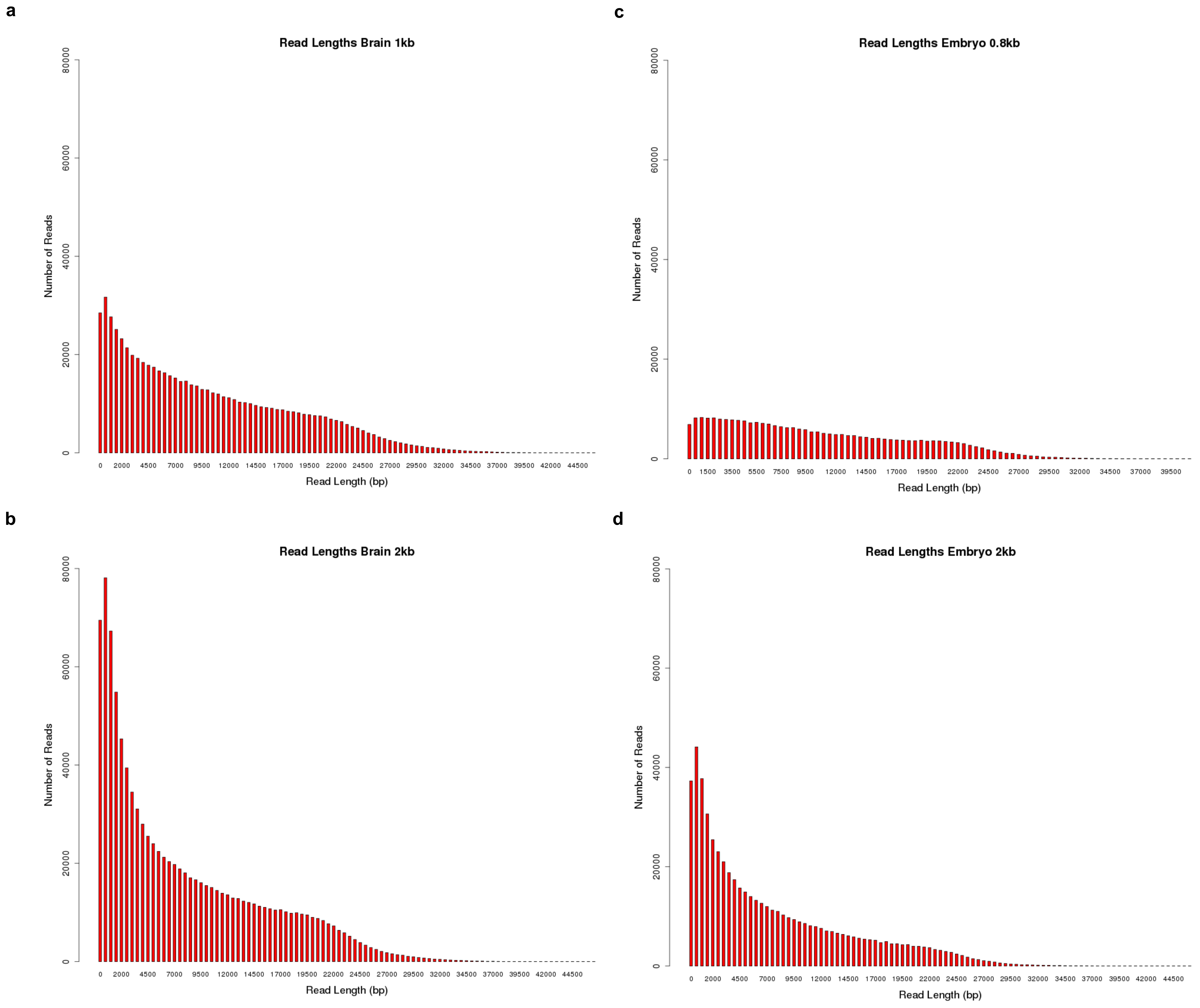

Additional File 1 | PacBio sequencing read lengths (**a**) Read lengths for chicken brain 1-kb size selection. (**b**) Read lengths for chicken brain 2-kb size selection. (**c**) Read lengths for chicken embryo 0.8-kb size selection. (**d**) Read lengths for chicken embryo 2-kb size selection.
